# Cysteine as an Alternative Eco-Friendly Corrosion Inhibitor for Absorption-Based Carbon Capture Plants

**DOI:** 10.3390/ma16093496

**Published:** 2023-05-01

**Authors:** Mohamed Ishaq Habibullah, Amornvadee Veawab

**Affiliations:** Faculty of Engineering and Applied Science, University of Regina, Saskatchewan, SK S4S 0A2, Canada; ishaqcecri@gmail.com

**Keywords:** carbon capture, CO_2_ absorption, corrosion inhibitor, cysteine, electrochemical, weight loss

## Abstract

Inorganic corrosion inhibitors are commonly applied to mitigate severe corrosion in absorption-based carbon capture plants. They are, however, not environmentally friendly, posing a health risk, harming the environment, and making chemical handling and disposal costly. Therefore, this study evaluated the corrosion inhibition performance of an amino acid, namely cysteine, with the aim of providing an eco-friendly alternative to the commercial inorganic corrosion inhibitors. Electrochemical and weight loss corrosion measurements showed that cysteine was effective in protecting carbon steel at all process operating conditions. At 80 °C, a 500 ppm cysteine could provide up to 83% and 99% inhibition efficiency under static and dynamic flow conditions, respectively. Its inhibition efficiency could be improved when the cysteine concentration, solution temperature, and flow condition were altered. Cysteine was an anodic corrosion inhibitor and underwent spontaneous, endothermic, and combined physical and chemical adsorption that followed the Langmuir adsorption isotherm model. The quantum chemical analysis indicated that cysteine had a high reactivity with metal surfaces due to its low energy gap and high dipole moment. The EDX analysis revealed a significant sulphur content on the metal substrate, indicating that cysteine’s mercapto group played an integral role in forming an effective adsorption layer on the metal interface.

## 1. Introduction

The use of corrosion inhibitors is a common practice for corrosion mitigation in the carbon dioxide (CO_2_) absorption process used for carbon capture from industrial waste gases. However, current corrosion inhibitors, such as vanadium pentoxide, sodium metavanadate, potassium antimonyl tartrate, bismuth nitrate, and nickel (II) sulphate hexahydrate, are toxic inorganics that pose a threat to the environment and public health [1,2,3]. Since many countries have imposed stricter environmental laws concerning toxic and hazardous substances, it is becoming necessary to find alternative corrosion inhibitors that are environmentally benign to replace current inorganic inhibitors. Numerous studies have been carried out over two decades to investigate the feasibility of using low-toxic inhibitors in the absorption-based carbon capture process [2,3,4,5,6,7]. Although no effective eco-friendly corrosion inhibitor has yet emerged as a viable alternative to the current inhibitors, the findings obtained from these previous studies seem to suggest that sulphur-containing organic corrosion inhibitors have potential to perform well in aqueous amine-CO_2_-O_2_ environments, and, thus, should be further explored. 

**Figure 1:**
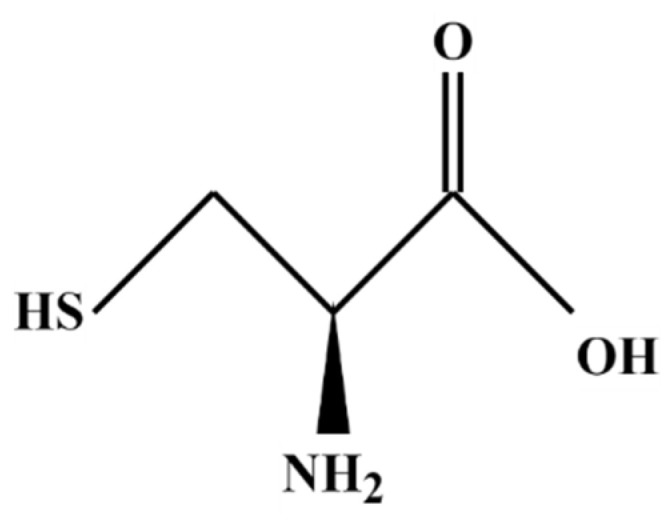
Chemical structure of cysteine.

This study evaluated the corrosion inhibitive behavior of cysteine (Cys), a sulphur-containing amino acid, on carbon steel 1018 in the CO_2_ absorption process using amine solvents. Cysteine was selected because of its environmentally friendly characteristics and its past performance in other environments. As illustrated in Figure 1, cysteine (C_3_H_7_NO_2_S) comprises mercapto (-SH), carboxyl (-COOH), and amine (-NH_2_) groups. It is essentially the main protein found in nails, hair, and skin, and is used for making collagen supplements, as well as being used as the precursor in food, and pharmaceutical and personal care products [8]. In comparison to the commonly used amine absorption solvents and inorganic corrosion inhibitors shown in Table 1, cysteine is not harmful and is easily biodegradable, as well as having a low potential to bioaccumulate in aquatic organisms. The lethal dose (LD_50_) value of cysteine based on rat (oral) is greater than those of amine solvents (e.g., MEA, DEA, and MDEA), and much greater than those of inorganic inhibitors (e.g., sodium metavanadate, copper carbonate, vanadium pentoxide, and nickel (II) sulfate hexahydrate) [9,10]. About 98% of cysteine can be degraded in an aerobic environment after 28 days. Such a biodegradation rate is comparable to MEA, but faster than MDEA [9,10,11]. The bioaccumulation of cysteine represented as a log partition in octanol/water (*pow*) is less than those of MEA, DEA, and MDEA. Sodium metavanadate, copper carbonate, vanadium pentoxide, and nickel (II) sulfate hexahydrate are toxic to aquatic life because of their high tendency of bioaccumulation [10]. The inhibition potential of cysteine has been exhibited by its past performance for various metals in many environments [12,13,14,15,16]. For instance, cysteine was reported to perform well in a CO_2_-saturated brine environment, yielding a 91% inhibition efficiency due to its sulphur atom contained in the mercapto group and nitrogen/oxygen atoms contained in the carboxyl group [16].

## 2. Materials and Methods

### 2.1. Tested Specimens and Absorption Solutions

All tested specimens were made of carbon steel 1018 consisting of 0.184% carbon, 0.011% manganese, 0.014% phosphorous, 0.170% sulphur, 0.010% silicon, 0.100% copper, 0.100% chromium, and balance iron. The cylindrical electrochemical specimen had 4.52 cm^2^ surface area with 0.6 cm inner diameter, 1.2 cm outer diameter, and 1.2 cm height. The weight loss specimen had flat, square shape having a dimension of 2.5 cm × 2.5 cm and 0.3 cm thickness. All specimens were polished with silicon carbide papers (up to 600 grit), degreased by acetone, ultrasonically cleaned for 5 min, and dried using warm air [17]. They were weighed before and after each experiment on a balance with ± 0.0001 g accuracy. Their surface morphology was characterized by the Fischer Scientific Nova Nano SEM 450 equipped with Octane Super EDX detector.

The amine solution selected for this study was 5.0 kmol/m^3^ (30 wt%) aqueous monoethanolamine (MEA) solution. It was prepared from deionized water and 90 wt% reagent grade MEA, and saturated with CO_2_ at room temperature (about 23 °C). Before and after the tests, the MEA solution was titrated with a 1N hydrochloric acid (HCl) using methyl orange indicator to determine its concentration and CO_2_ loading.

### 2.2. Electrochemical Measurements

Electrochemical corrosion measurements were performed in a 100 mL jacketed microcell having a graphite counter electrode and a silver/silver chloride reference electrode. The microcell was immersed in a temperature-controlled water bath for maintaining solution temperature and connected to a speed-controlled rotor for controlling an angular rotational speed of the working electrode. It was then fitted with a gas disperser for CO_2_ and O_2_ supply, and a condenser for preventing vapor loss of the MEA solution. A 263A potentiostat (Princeton Applied Research, Oak Ridge, TN, USA) was equipped with a 5210 lock-in amplifier for electrochemical scans and electrochemical response monitoring. The data acquisition unit functioned as a hub for data recording and data analysis with the help of the Powersuite Software version 2.53.

During the experiment, the MEA solution saturated with CO_2_ was purged with 85% CO_2_ and 15% O_2_ gas to maintain CO_2_ saturation and simulate O_2_ content that typically exists in waste gas streams. To mimic the solution’s typical linear velocity of the lean and rich amine piping (0.9 m/s) [1], the rotor was set to run at a 1500 rpm rotational speed. The electrochemical experiment was started by measuring the system’s Open Circuit Potential (OCP) at a potential variation of less than 3 mV/min [18,19]. A series of electrochemical scans were then performed successively, beginning with Electrochemical Impedance Spectroscopy (EIS), followed by Linear Polarization Resistance (LPR), and then Potentiodynamic Polarization (PP). The EIS scan was recorded after the specimen was immersed in the solution for a minimum of 6 h for OCP stabilization. The LPR scan was recorded after 45 min of the EIS scan, while the PP scan was begun right after the LPR scan. Each electrochemical scan was replicated.

The EIS scan was conducted from 10 mHz to 10kHz while the LPR scan was done at ±20 mV vs. the OCP. The inhibition efficiency of cysteine (*η*) was determined from the obtained EIS and LPR data using the following equation [20,21].

(1) $$\eta =\{1-\frac{R_{p}^{0}}{R_{p}}\}$$

where *R_p_* denotes the polarization resistance of the cysteine system (Ω cm^2^) and $R_{p}^{0}$ represents the polarization resistance of the uninhibited system (Ω cm^2^). The PP scan was performed from −0.25 V to 1.2 V vs. the OCP at 0.166 mV/s. Following the Tafel extrapolation method [21], the *η* from PP scans was assessed by using Equation (2) [22].

(2) $$\eta =(\frac{i_{corr}^{0}-i_{corr}}{i_{corr}^{0}})$$

where *i^0^_corr_* is the uninhibited corrosion current density and *i_corr_* is the inhibited corrosion current density. 

### 2.3. Weight Loss Corrosion Measurements

The weight loss corrosion measurements were implemented using a 5 L autoclave containing a 5.0 kmol/m^3^ MEA solution saturated with CO_2_. Four specimens were hung inside the autoclave at four locations. Specimen M1 was fully immersed in liquid under a dynamic flow condition. Specimen M2 was also fully immersed in liquid, but under a static condition. Specimen M3 was partially exposed to liquid and vapor under the static condition. Specimen M4 was fully exposed to vapor under the static condition. Similarly to the electrochemical measurements, a gas mixture containing 85% CO_2_ and 15% O_2_ was introduced to the MEA solution at 5 bar and 120 °C. After 21 days of exposure, all tested specimens were taken out from the autoclave and underwent a cleaning process in accordance with the ASTM Standard G1-03e1 [17]. The tested specimens were immersed in distilled water for ultrasonic cleaning for about 5 min to remove corrosion products from the specimen surface and were then degreased in an ultrasonicator containing acetone for 2–3 min. They were subsequently cleaned using Clark’s solution for 1–2 min to remove residues, dried using warm air, and, finally, weighed. The corrosion rate of each specimen was estimated from Equation (3).

(3) $$CR=\frac{87.6\;W}{DAT}$$

where *CR* is the rate of corrosion (mmpy), *W* is the weight loss (mg), *D* is the density of metal (g/cm^3^), *A* is the metal’s exposure area (cm^2^), and *T* is the exposure duration (hour). The *η* of cysteine was calculated using Equation (4) [23], where *CR*^0^ and *CR* represent corrosion rates in blank and cysteine-inhibited systems, respectively.

(4) $$\eta =\left(\frac{{CR}^{0}-CR}{{CR}^{0}}\right)$$

## 3. Results and Discussion

### 3.1. Electrochemical Inhibition Performance

#### 3.1.1. Effect of Cysteine Concentration

**Figure 2:**
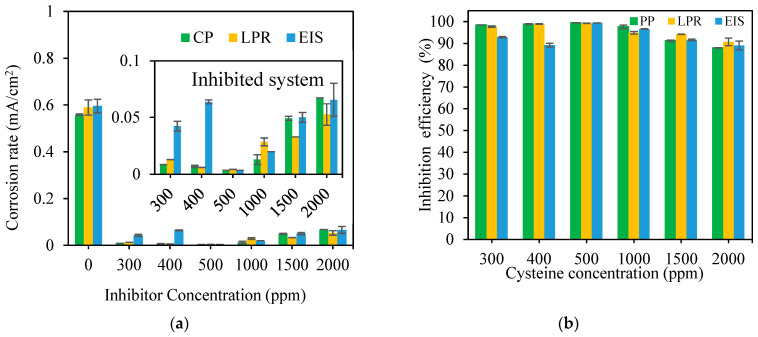
Effect of cysteine concentration on: (**a**) corrosion rate; (**b**) inhibition efficiency (80 °C and 1500 rpm).

The effect of the cysteine concentration on the inhibition performance was evaluated using 300, 400, 500, 1000, 1500, and 2000 ppm cysteine at 80 °C under the dynamic flow condition simulated by 1500 rpm rotational speed. Figure 2 shows comparable corrosion rates obtained from PP, LPR, and EIS scans. The existence of cysteine significantly reduced the corrosion rates of the blank (uninhibited) system. For example, when 500 ppm cysteine was added, the corrosion rate was reduced from 0.5810 to 0.0037 mA/cm^2^, with an inhibition efficiency of 99.34%.

The results also showed that the cysteine concentration had an apparent effect on the inhibition performance. Raising the cysteine concentration from 300 to 500 ppm improved the inhibition efficiency from 96.33% ± 0.21% to 99.34% ± 0.04%. However, a further increase in the cysteine concentration to 2000 ppm reduced the inhibition efficiency to 89.24% ± 1.27%. This might be due to the hemi-micelle development, or in this case, the monolayer of cysteine attaching to the metal interface. When the cysteine hemi-micelles exceeded its critical concentration at 500 ppm, some cysteine molecules might have been desorbed from the interface [16,24]. The critical concentration of 500 ppm could be considered as the optimum cysteine concentration, yielding the highest inhibition efficiency at the minimum inhibitor concentration and cost.

**Figure 3:**
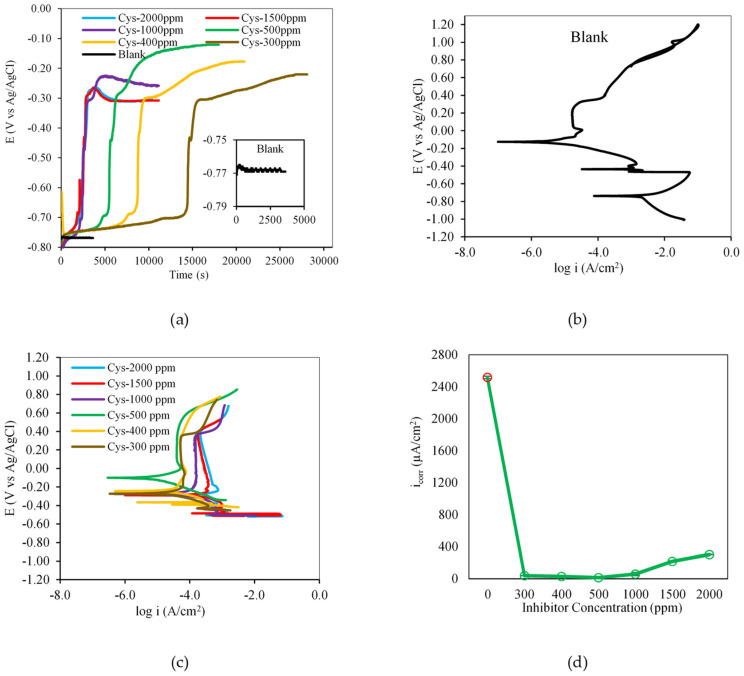
Effect of cysteine concentration on: (**a**) open circuit potential; (**b**) potentiodynamic polarization behavior of the blank system; (**c**) potentiodynamic polarization behavior of the cysteineinhibited systems; (**d**) corrosion current density (80 °C and 1500 rpm).

The inhibition effectiveness of cysteine was evident in the obtained electrochemical data. From Figure 3a, it can be seen that the presence of cysteine largely shifted the system’s OCP to a more noble range. That is, the OCP increased from −0.767 V in the blank system to above −0.300 V when 300–2000 ppm cysteine was introduced to the MEA solution. This indicates that the presence of cysteine led to the adsorption of cysteine molecules onto the metal surface, which changed the composition and structure of the electrical double layer and formed an inhibitive adsorption layer on the metal–solution interface [25]. The cysteine adsorption appeared to progress in three stages. The first stage took place when the carbon steel specimen was first in contact with the solution at the onset of the OCP measurement. The adsorption of cysteine molecules onto the specimen surface was initiated at a slow rate. The second stage exhibited a sudden rise in the system’s potential. This suggests a fast cysteine adsorption rate with a drastic increase in the number of adsorbed cysteine on the metal interface, providing more coverage of the protective cysteine complex layer. The third stage was reached when the potential became more stabilized with time. In this final stage, the cysteine adsorption rate was slowed down to the point where little or no further cysteine molecules were adsorbed onto the metal surface, the maximum inhibitor coverage was reached, and the metal–solution interface entered the equilibrium state.

Figure 3a also shows that more time was required for the cysteine-inhibited system to reach an equilibrium state at the OCP. The OCP of the blank system was attained quickly at about 500 s, while the OCP of the system containing 300–2000 ppm cysteine was reached at 10,000–30,000 s. This suggests that cysteine took time to interact with carbon steel to develop corrosion resistance and move into a new state of the system’s equilibrium. The time to reach the OCP was, however, reduced from 30,000 s to 10,000 s once the cysteine concentration was increased from 300 to 2000 ppm. This was due to the increasing number of cysteine molecules that were available for adsorption to form a protective layer.

Figure 3b illustrates the polarization characteristics of the tested carbon steel in the uninhibited MEA solution. The carbon steel underwent an active–passive transition with the intersection of the cathodic polarization curve at the corrosion potential (*E_corr_*) of −762.55 ± 1.23 mV in the active region. In this region, the corrosion process was undergone with no passivation. The passivation could take place when the potential exceeded the primary passive potential at about −0.5 V. The passive film was, however, not stable. It tended to break down and repassivate at about −0.4 V and −0.1 V, creating a couple of anodic noses. The carbon steel then proceeded into the transpassive region at about 0.3 V. 

In the cysteine-inhibited system, the formation of a corrosion-resistant layer was evidenced by potentiodynamic polarization curves. As illustrated in Figure 3c, the carbon steel specimens underwent spontaneous passivation with the *E_corr_* of −302.85 ± 8.20 mV and −103.79 ± 3.34 mV when 2000 ppm and 500 ppm of cysteine, respectively, were present in the MEA solutions. The *E_corr_* values of the cysteine-inhibited system were more than 450 mV higher than those of the blank system. This implies that cysteine functioned as an anodic-type inhibitor [26] which primarily retarded the metal dissolution at the anodic site, which in turn reduced the corrosion current density (*i_corr_*). For example, from Figure 3d, it can be seen that the value of *i_corr_* was reduced drastically from 2515.5 ± 15.5 µA/cm^2^ in the blank system to 14.11 ± 1.24 µA/cm^2^ when 500 ppm cysteine was available in the solution. This suggests that cysteine was adsorbed onto the carbon steel, leading to the anodic shift, developing a passive layer, and reducing corrosion with its inhibitive effect. 

**Figure 4:**
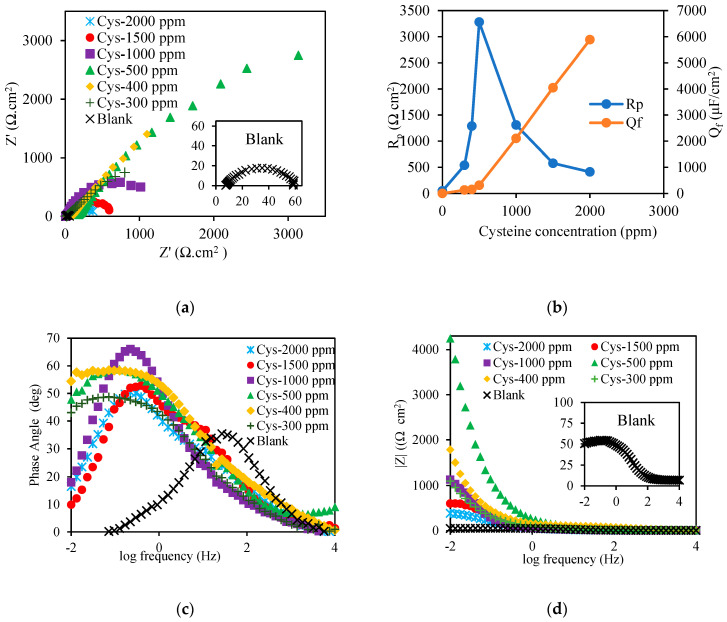
Effect of cysteine concentration on: (**a**) Nyquist plot; (**b**) polarization resistance and capacitance; (**c**) Bode plot; (**d**) modulus plot (80 °C and 1500 rpm).

To reveal the interaction between cysteine and the metal interface, the obtained EIS data were analyzed. From Figure 4a, the Nyquist Plot showed a depressed semi-circle for the blank system and partial semi-circles for the cysteine-inhibited system. The depressed semicircle in the blank system depicted the non-ideal capacitive behavior of the system’s interface due to the metal surface’s inhomogeneity caused by electrode porosity, surface disorder, and roughness [27]. In the cysteine-inhibited system, capacitive loops possessed imperfect semicircles, suggesting that the metal surface was heterogeneous due to uneven charge distribution at the metal–electrolyte interface [25]. 

In addition, Figure 4a illustrates that the cysteine concentration affected the polarization resistance (*R_p_*). Raising the cysteine concentration from 300 to 500 ppm increased the diameter of the Nyquist plot, thereby increasing *R_p_* from 539.16 ± 19.49 Ω cm^2^ to 3279.33 ± 53.63 Ω cm^2^. This might be due to the enhancement of cysteine’s interfacial coverage on carbon steel [28]. However, the further increase in the cysteine concentration from 500 ppm to 2000 ppm resulted in a decrease in the Nyquist plot’s diameter, thus reducing *R_p_* to 412.91 ± 5.82 Ω cm^2^. 

As exhibited in Figure 4b, the film capacitance of the constant phase element (*Q_f_*) increased considerably from 312.21 ± 16.24 μF/cm^2^ to 5884.50 ± 2.13 μF/cm^2^ as a result of increasing the cysteine concentration from 500 to 2000 ppm. This suggests that using cysteine with concentrations greater than 500 ppm could cause disintegration of the inhibitor film at the electrical double layer and reduce the effectiveness of cysteine. 

The impedance data were further analyzed as the Bode plot in Figure 4c. It was apparent that the cysteine-inhibited system had higher phase angle values than the blank system with a single time constant. The maximum phase angle of the cysteine-inhibited system was 65°, corresponding to capacitive behavior, while the maximum phase angle of the blank system was 35°, corresponding to diffusive behavior [29]. The phase angle increased when the cysteine concentration was increased from 300 to 1000 ppm due to the formation of a more compact protective layer with smaller pore sizes which could block the passage of corrosive species [30]. 

Figure 4d illustrates the Modulus plots of the cysteine-inhibited systems with the constant impedance for when the log frequency was in the range of 1–4 Hz. This confirmed that the total impedance was solely due to solution resistance and capacitor. The absolute impedance was improved to the maximum of 4248.25 Ω cm^2^ when the cysteine concentration was increased from 300 to 500 ppm. However, it decreased to the minimum of 386.08 Ω cm^2^ when the cysteine concentration was further raised to 2000 ppm. This implies that the capacitor’s impedance was greater than the impedance of polarization resistance at a low frequency, preventing the corrosion current passing through the capacitor [3]. Such capacitive behavior illustrated that the corrosion inhibition occurred in the presence of cysteine.

**Figure 5:**
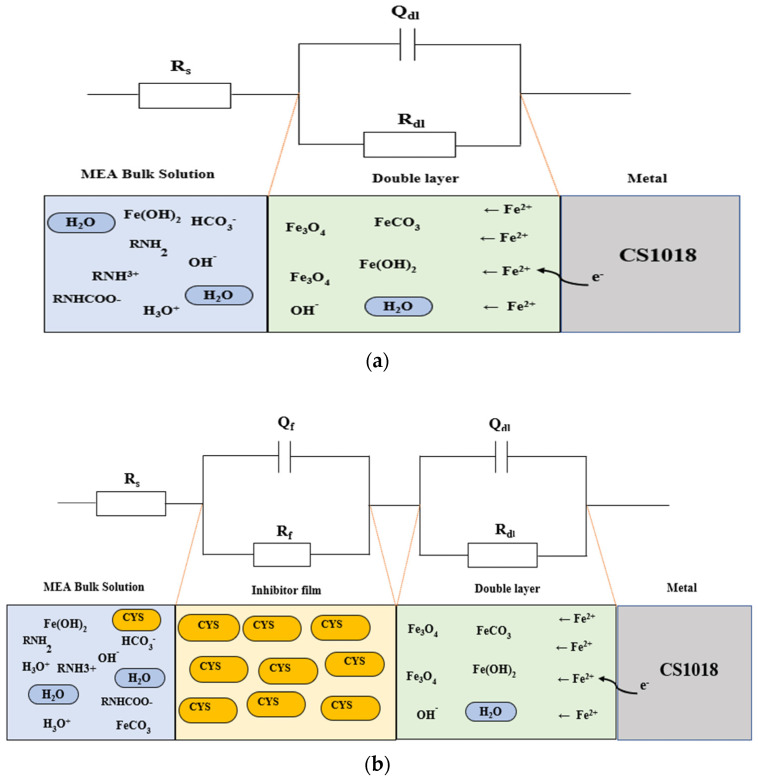
Illustration of the equivalent circuit of CS 1018−MEA interface of: (**a**) blank system; (**b**) cysteine−inhibited system.

To further reveal the inhibition mechanism of cysteine, the ZSimpWin software (Ametek SI, Berwyn, PA, USA) was used for modeling the equivalent circuit of the blank and cysteine systems. At 80 °C and 1500 rpm, the *R*(*QR*) equivalent circuit (Figure 5a) was best-fitted for the blank system with a 3.2% error, while the *R*(*QR*)(*QR*) equivalent circuit (Figure 5b) was best-fitted for the system containing 500 ppm cysteine with a 1.6% error. The *R*(*QR*) equivalent circuit of the blank system represented a train of solution resistance (*R_s_*) and double-layer resistance (*R_dl_*) (or polarization resistance (*R_p_*)) which was parallel to the double layer capacitance (*Q_dl_*) (or constant phase element (CPE)). This circuit indicated the charge transfer between the bulk solution and carbon steel surface through the double-layer interface. The blank system had an *R_s_* of 7.55 ± 0.14 Ω cm^2^, *R_dl_* (or *R_p_*) of 50.76 ± 1.85 Ω cm^2^, *Q_dl_* of 1234.5 ± 14.5 μF/cm^2^, and *n_dl_* of 0.78 ± 0.02. The value of *n_dl_* approaching the unity suggested that the blank system may function as a capacitor, but not an ideal capacitor.

The equivalent circuit for the cysteine-inhibited system was made up of a series of *R_s_*, *Q_f_R_f_*, and *Q_dl_R_dl_*. *R_f_*, representing film resistance, and *Q_f_*, representing film capacitance, were connected in parallel. The cysteine film layer was a great addition to the blank system as it provided an incremental resistance (*R_f_*) in the cysteine-inhibited system. For example, the value of *R_p_* increased from 50.76 ± 1.85 Ω cm^2^ in the blank system to 3279.33 ± 53.63 Ω cm^2^ in the 500 ppm cysteine system, which was more than 60 times greater than the blank. The increase in *R_p_* retarded the corrosion process, thus leading to the decrease in corrosion rate. Furthermore, the inhibitor film reduced the *Q_f_* from 1234.50 ± 14.50 μF/cm^2^ in the blank system to 312.21 ± 16.24 μF/cm^2^ in the 500 ppm cysteine system. This may have resulted from the thickening of an inhibitor film and the effective integration with the double layer [31]. 

#### 3.1.2. Effect of Temperature 

**Figure 6:**
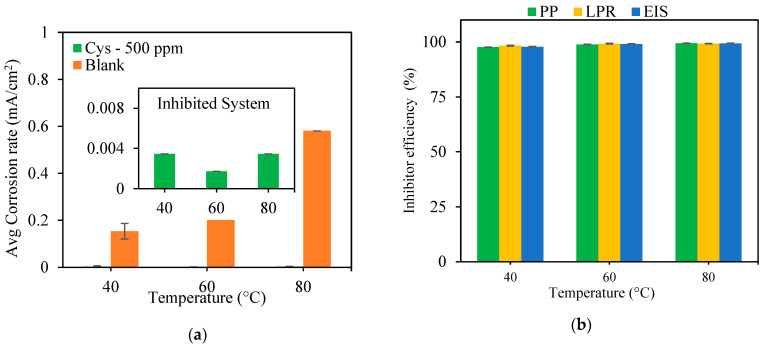
Effect of solution temperature on: (**a**) corrosion rate; (**b**) inhibition efficiency (500 ppm cysteine and 1500 rpm rotational speed).

The temperature effect on cysteine’s performance was evaluated for the MEA solution containing 500 ppm cysteine at a 1500 rpm rotational speed. The results in Figure 6a indicate that the solution temperature had an influence on the corrosion rates of both the blank and cysteine-inhibited systems. The corrosion rate of the blank system increased with the increase in temperature from 40 °C to 80 °C. This was attributed to the increases in the rates of anodic and cathodic reactions. In the cysteine-inhibited system, the corrosion rate decreased when the solution temperature was increased from 40 °C to 60 °C due to the enhancement of cysteine chemisorption onto the metal surface. The further rise of the solution temperature from 60 °C to 80 °C, however, increased the corrosion rate of the inhibited system. This might be because the rise in temperature to 80 °C led to the increasing amounts of corrosive species available for cathodic reactions and also weakened the electrostatic interaction between the cysteine molecules and the metal interface, which could result in cysteine desorption. In taking into account the corrosion rates of both the blank and inhibited systems, the inhibition efficiency of cysteine slightly increased from 97.82 ± 0.07% at 40 °C to 99.34 ± 0.04% at 80 °C (Figure 6b). 

**Figure 7:**
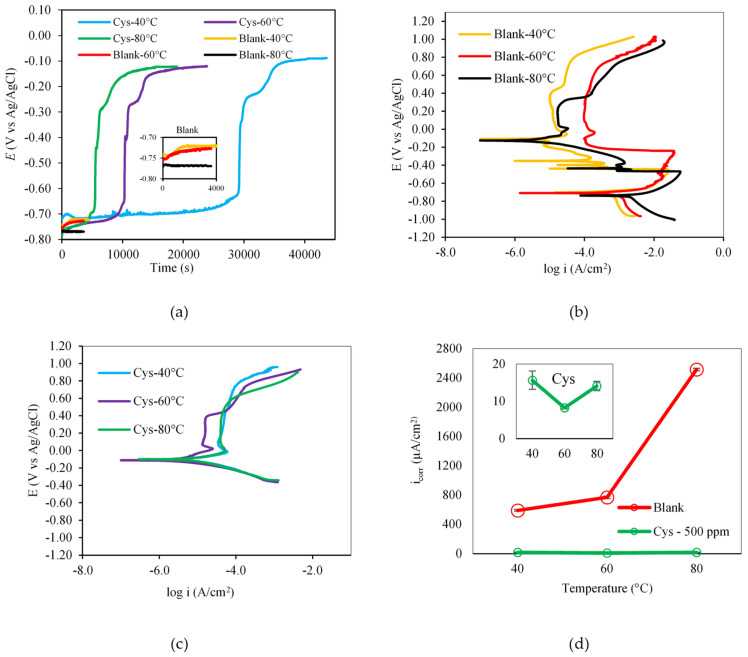
Effect of solution temperature on (**a**) open circuit potential; (**b**) potentiodynamic polarization behavior of the blank system; (**c**) potentiodynamic polarization behavior of the cysteine–inhibited systems; (**d**) corrosion current density (500 ppm cysteine and 1500 rpm rotational speed).

Figure 7a shows that the solution temperature had an influence on the time taken for the cysteine-inhibited system to reach equilibrium. As the temperature was increased from 40 °C to 60 °C and 80 °C, the time to establish OCP decreased from 40,000 s to 22,000 and 15,000 s, respectively. This indicated that, at a lower temperature, cysteine took more time to develop an adsorption layer on the carbon steel surface, while the higher temperature promoted the adsorption rate of cysteine on CS 1018. 

The potentiodynamic polarization curves in Figure 7b indicate that, in the blank solution, the carbon steel underwent an active–passive transition with three different regions (active, passive, and transpassive). It appeared to remain in the active state at all tested temperatures. Raising the solution temperature caused both the anodic and cathodic polarization curves to shift toward higher current densities, indicating higher rates of iron oxidation and corroding agent reduction. For the cysteine-inhibited system, the carbon steel was in the passive state at all of the tested temperatures (Figure 7c). The temperature variation primarily shifted the anodic polarization curves, affecting the iron dissolution rate at the anodic sites. The *E_corr_* values of the cysteine system were at a more noble potential range of −0.100 V, while those of the blank system ranged from −0.720 to −0.760 V. The shift in *E_corr_* values of greater than 85 mV suggested that cysteine acted as an anodic-type inhibitor at all of the tested temperatures. This implies that the corrosion inhibition by cysteine predominantly occurs at the anodic site, not the cathodic site. Figure 7d also shows that the *i_corr_* values of the cysteine-inhibited system were much lower than those of the blank system for all of the tested temperatures, thus indicating the inhibitive performance of cysteine. 

**Figure 8:**
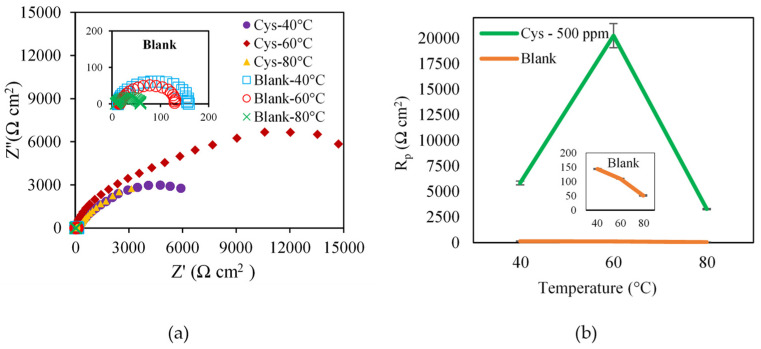
Effect of solution temperature on: (**a**) Nyquist Plot; (**b**) polarization resistance (500 ppm cysteine and 1500 rpm rotational speed).

The Nyquist plots of the blank system (Figure 8a) illustrate the depressed semicircle impedance behavior with one time constant, indicating that the corrosion was charge-transfer controlled [32]. The partial semicircles of the cysteine system suggested capacitive behavior involving double layer capacitance. As shown in Figure 8b, the *R_p_* values of the blank system decreased from 143.7 ± 1.5 Ω cm^2^ to 50.76 ± 1.85 Ω cm^2^ when the temperature was raised from 40 °C to 80 °C. This may be attributed to the increasing mass transport of both ferrous ions and corroding agents to and from the metal surface. In contrast, the *R_p_* values of the cysteine-inhibited system increased from 5829.92 ± 184.84 Ω cm^2^ at 40 °C to 20238.5 ± 1178.5 Ω cm^2^ at 60 °C due to the promotion and strength of the cysteine chemisorption on the metal surface. A further increase in the solution temperature to 80 °C, however, resulted in a decrease of the *R_p_* value to 3279.55 ± 53.63 Ω cm^2^. This may be because the rise in temperature caused some cysteine molecules to desorb from the metal interface, especially when cysteine underwent physical adsorption with weak electrostatic force.

#### 3.1.3. Effect of Flow Condition 

**Figure 9:**
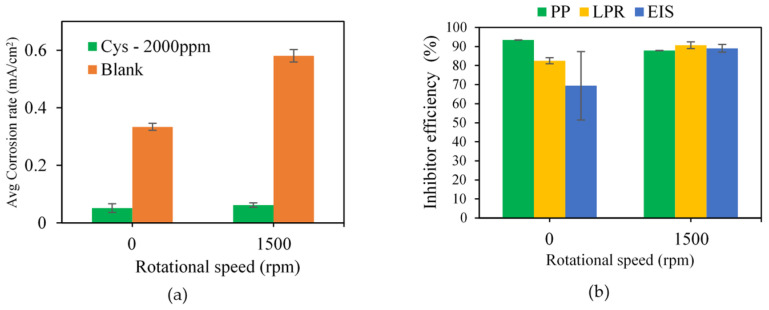
Effect of rotational speed on: (**a**) corrosion rate; (**b**) inhibition efficiency (2000 ppm cysteine 80 °C).

The effect of the flow condition on cysteine’s performance was evaluated by varying the rotational speed of the working electrodes from 0 rpm, representing the static condition, to 1500 rpm, representing the dynamic condition, for the MEA solution containing 2000 ppm cysteine at 80 °C. The electrochemical results in Figure 9a illustrate that increasing the rotational speed from 0 to 1500 rpm increased the corrosion rate from 0.33 ± 0.01 mA/cm^2^ to 0.58 ± 0.02 mA/cm^2^ in the blank system, and from 0.05 ± 0.01 mA/cm^2^ to 0.06 ± 0.01 mA/cm^2^ in the cysteine-inhibited system. The dynamic condition in the blank system may have increased the mass transport of ferrous ion (Fe^2+^) from the metal surface to the bulk solution, thus resulting in less protective corrosion products (e.g., iron carbonate) being present at the metal–solution interface. In the cysteine-inhibited system, the dynamic condition may have caused a high shear stress, which could lead to desorption of some cysteine–metal complex from the metal surface. Overall, cysteine performed well considering the inhibition efficiencies of 81.78 ± 6.56% under the static condition and 89.24 ± 1.27% under the dynamic condition (Figure 9b). A slight increase in the inhibition efficiency in the dynamic condition might be due to the improvement of the cysteine transport, providing more cysteine molecules at the metal surface for adsorption. 

**Figure 10:**
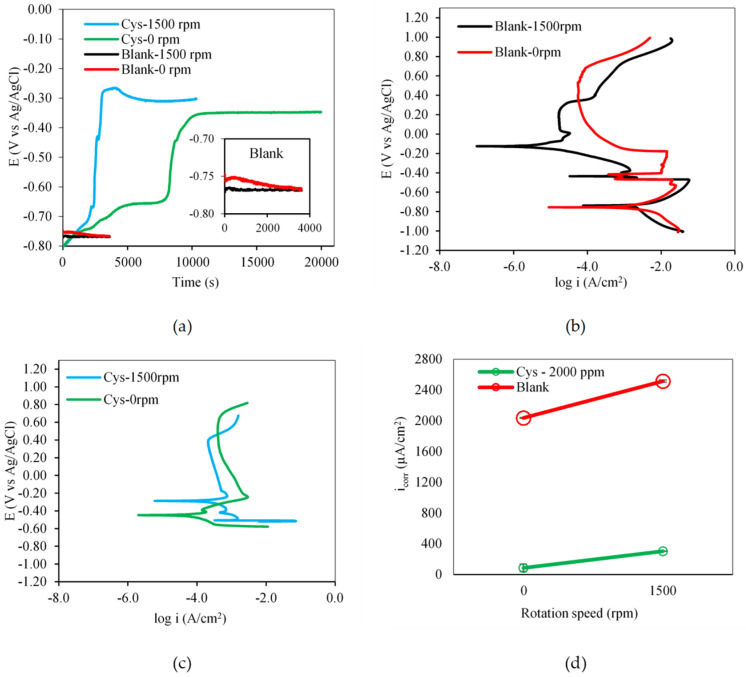
Effect of rotational speed on: (**a**) open circuit potential; (**b**) potentiodynamic polarization behavior of the blank system; (**c**) potentiodynamic polarization behavior of the cysteine−inhibited systems; (**d**) corrosion current density (2000 ppm cysteine and 80 °C).

Figure 10a shows that the flow condition had a great impact on the time for a system to reach equilibrium. The dynamic flow condition enabled the cysteine-inhibited systems to achieve OCP more quickly compared to the static condition. This was because the dynamic condition increased the mass transfer rate of chemical species between the MEA solution and the carbon steel surface, and facilitated the mass transport during the adsorption of cysteine molecules onto the metal interface [13].

The polarization curves of the blank solution in Figure 10b exhibited an active–passive transition. The carbon steel specimens were in the active state at both the static and dynamic conditions. Figure 10c illustrates the passive state of the specimens in the cysteine-inhibited system. The presence of the cysteine adsorptive layer retarded the transport of reacting species and products at the metal–solution interface and thus reduced *i_corr_* (Figure 10d).

**Figure 11:**
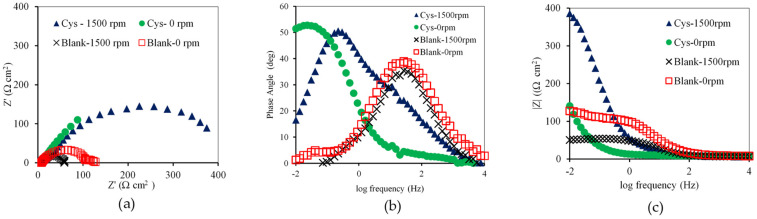
Effect of rotational speed on: (**a**) Nyquist Plot; (**b**) Bode plot; (**c**) modulus Plot (2000 ppm cysteine and 80 °C).

The flow condition did not affect the impedance behavior of the blank and cysteine-inhibited systems. Figure 11a, under both static and dynamic conditions, shows the semi-circle Nyquist plots which were obtained for the blank system and the partial semi-circle plots which were apparent for the cysteine-inhibited systems. Such partial semicircle impedance behavior may have been a result of an inhomogeneous carbon steel surface [33,34]. The *R_p_* increased from 112.43 ± 1.83 Ω cm^2^ in the blank system to 343.66 ± 3.9 Ω cm^2^ in the cysteine-inhibited system under the static condition. Similarly, it also increased from 50.76 ± 1.85 Ω cm^2^ in the blank system to 412.91 ± 5.82 Ω cm^2^ in the cysteine-inhibited system under the dynamic condition. 

The Bode plot (Figure 11b) illustrates that the phase angle of the blank system was in the range of 35° to 38° with the resistive behavior, while that of the cysteine-inhibited system was in the range of 50° to 52°, reflecting the capacitive behavior. The increase in the phase angle suggested that cysteine forms a passive film on the metal surface, hindering the interaction with corrosive species [31]. The change in rotational speed from static to dynamic conditions did not cause much variation in the phase angle. This indicated that cysteine was effective under both the static and dynamic conditions. This result was supported by the modulus plot in Figure 11c, in which the absolute impedance of the cysteine system was found to be higher than that of the blank system under the static and dynamic flow conditions. 

#### 3.1.4. Post Analysis

The adsorption isotherm was analyzed to reflect the cysteine concentration effect on inhibition effectiveness. The analysis was done by fitting the obtained electrochemical data to the expression of four adsorption equilibrium models, namely Langmuir, Temkin, Frumkin, and El-Awady. The mathematical expressions of these models are given below.

Langmuir isotherm [35]:

(5) $$\frac{C}{\theta }=\frac{1}{K_{ads}}+C$$

Temkin isotherm [36]:

(6) $$\theta =\frac{-lnC}{2\alpha }-\frac{lnK_{ads}}{2\alpha }$$

Frumkin isotherm [36]:

(7) $$\log\frac{\theta }{1-\theta }\cdot C=\log K_{ads}+\frac{2\alpha \theta }{2.303}$$

El-Awady isotherm [36]:

(8) $$log\frac{\theta }{1-\theta }=\log K+ylogC$$

where *C*, θ, *K_ads_*, *α*, and *K*, denote the cysteine concentration (mM), inhibition efficiency of cysteine, adsorption equilibrium constant (mM^−1^), adsorbate interaction factor, constant related to *K_ads_* (*K_ads_ = K*^1*/y*^), and number of cysteine molecules adsorbed onto carbon steel, respectively. 

**Figure 12:**
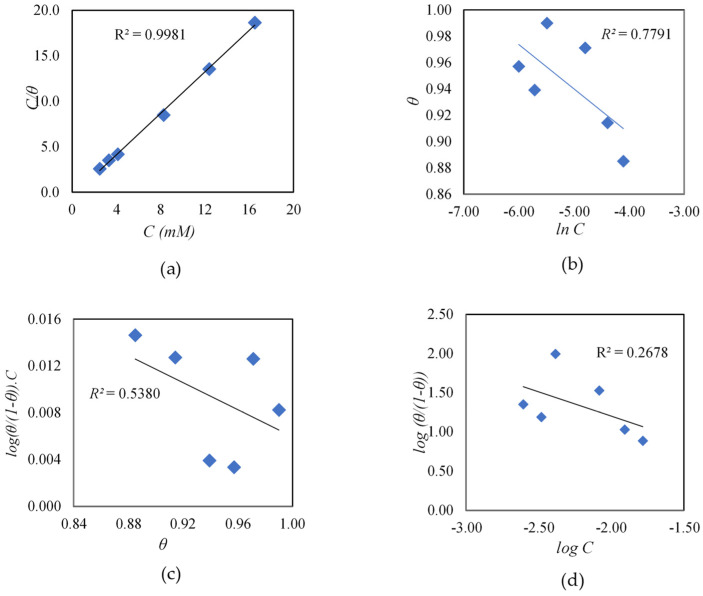
Adsorption isotherm plots of cysteine−inhibited system: (**a**) Langmuir; (**b**) Temkin; (**c**) Frumkin; (**d**) El-Awady (80 °C and 1500 rpm rotational speed).

From Figure 12, it can be seen that the Langmuir isotherm expression was the best fit to the experimental electrochemical data, with an *R*^2^ of 0.9981, compared to the Temkin, Frumkin, and El-Awady isotherms with *R*^2^ values of 0.7791, 0.5380, and 0.2678, respectively. This indicated that the adsorption of cysteine followed the Langmuir isotherm, which established a monolayer on the carbon steel surface with no interaction among the adsorbed cysteine molecules [37]. Using the intercept of the Langmuir plot and Equation (9) [35,36], the values of *K_ads_* and the standard Gibbs free energy change of adsorption (Δ*G°_ads_*) for cysteine were estimated to be 225.73 mM^−1^ and −27.70 kJ mol^−1^, respectively.

(9) $${\Delta G{}^{\circ}}_{ads}=-RT\;ln(55.5\;K_{ads})$$

where *R* and *T* represent the universal gas constant (8.314 J kmol^−1^) and temperature (Kelvin), respectively. The negative value of Δ*G°_ads_* indicates the spontaneous adsorption of cysteine onto carbon steel surface [36]. The Δ*G°_ads_* value in between −20 and −40 kJ mol^−1^ suggests that cysteine may undergo predominantly physical adsorption together with chemical adsorption [38]. 

**Figure 13:**
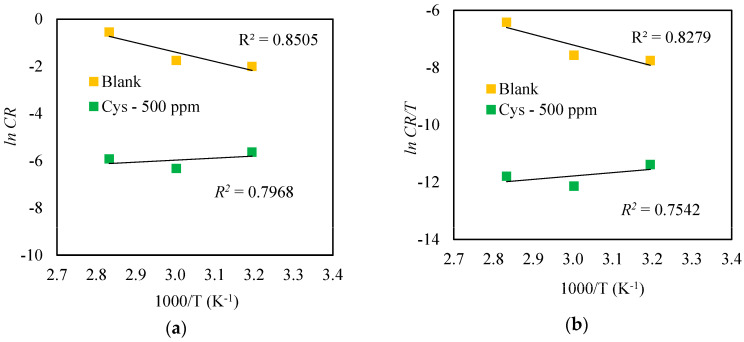
Temperature dependence of the cysteine−inhibited system: (**a**) Arrhenius plots; (**b**) transition state plots.

In addition to the adsorption isotherm, important temperature-dependent parameters were also analyzed from the obtained electrochemical data. The activation energy (*E_a_*) was determined from the Arrhenius expression in Equation (10) and the Arrhenius plots in Figure 13a.

(10) $$CR=A\;exp\frac{{-E}_{a}}{RT}$$

where *CR*, *A*, and *E_a_* denote the rate of corrosion (mg cm^−2^ h^−1^), pre-exponential factor of Arrhenius (mg cm^−2^ h^−1^), and apparent activation energy (kJ mol^−1^), respectively. The enthalpy (Δ*H_a_*) and entropy (Δ*S_a_*) of activation were assessed from the transition state equation below and the transition state plots in Figure 13b.

(11) $$CR=\frac{RT}{Nh}\exp\left(\frac{{\Delta S}_{a}}{R}\right)\exp\left(\frac{{\Delta H}_{a}}{RT}\right)$$

where *h*, *N*, Δ*H_a_*, and Δ*S_a_* denote Plank’s constant (6.626 × 10^−34^ Js^−1^), Avogadro’s number (6.023 × 10^23^ mol^−1^), the change in enthalpy of activation (kJ mol^−1^), and the change in entropy (J mol^−1^ K^−1^), respectively.

The results in Table 2 show that the *E_a_* and Δ*H_a_* of the cysteine-inhibited system were less than those of the blank system. This suggests that the cysteine underwent chemisorption, forming a chemical bond with the metal surface [39]. The positive value of Δ*H_a_* indicates an endothermic adsorption. The existence of cysteine in the MEA solution caused Δ*S_a_* to become more negative, reflecting spontaneous, endothermic adsorption of the cysteine [40].

### 3.2. Weight Loss Inhibition Performance 

**Figure 14:**
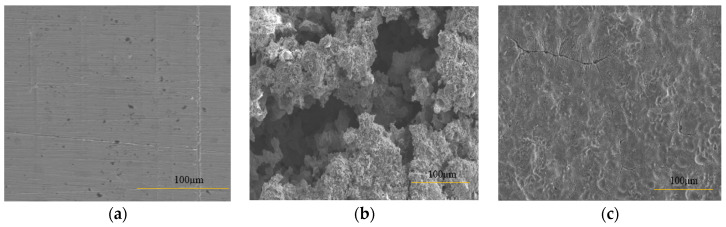
SEM images of CS 1018 specimens at M1 position: (**a**) before test; (**b**) blank system after test; (**c**) 500 ppm cysteine after test.

**Figure 15:**
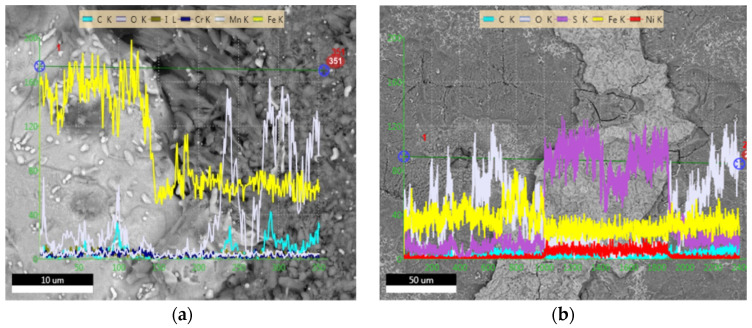
EDX line scan element profile plots of CS 1018 specimens after 21-day exposure at M1 position: (**a**) blank; (**b**) 500 cysteine.

The weight loss experiments were carried out for 21 days at 120 °C to determine the inhibitory performance of cysteine at the service temperature of a regenerator and reboiler, the hottest units in the amine-based absorption process. The results in Table 3 show that, in the absence of cysteine, carbon steel specimen M1 had the highest corrosion rate, followed by M2, M3, and M4. This suggested that the liquid phase was more corrosive to carbon steel than the liquid-vapor and vapor phases. The dynamic environment was more corrosive than the static. In the presence of cysteine, the corrosion rates of all specimens were significantly reduced to the range of 0.02–0.30 mmpy with the inhibition efficiency of 81–98%. Cysteine appeared to contribute to the improved surface morphology of the specimens in both the static and dynamic conditions. From the SEM images in Figure 14, it can be seen that the carbon steel surface from the cysteine-inhibited system was smoother, with much less corrosion damage, cracks, and pits compared to the uninhibited specimens. The EDX spectra taken from the steel surface in Figure 15 suggest the existence of cysteine on the tested carbon steel. The steel surface from the uninhibited system had major traces of the elements iron (Fe), carbon (C), oxygen (O), manganese (Mn), chloride (Cl), iodine (I), and chromium (Cr), whereas that of the cysteine system had sulfur (S) as an additional element.

### 3.3. Quantum Chemical Analysis

The quantum chemical analysis using the Density Functional Theory (DFT) with the basis set (6-311G++dp) was performed with the help of the Gaussian View-06 software. It was conducted to determine quantum electronic properties of cysteine, and revealed potential interaction between cysteine and metal, as well as reflecting corrosion inhibition ability. The analysis was not only conducted for cysteine, but also for methionine (MTI), the amino acid corrosion inhibitor which was tested in the most recent published work [7] for the purpose of performance comparison. It was found that the obtained quantum electronic properties reflect a high inhibition performance of cysteine, which aligns with the findings derived from electrochemical and weight loss experiments. The energy gap of cysteine (4.2611 eV) is less than that of methionine (5.5253 eV), suggesting that cysteine requires lower excitation energy to establish cysteine adsorption on a metal interface [41] compared to methionine. The dipole moment of cysteine (10.4479 Debye) is higher than that of methionine (2.6071 Debye). This indicates that cysteine is more polar than methionine, thereby having a great tendency to form a dipole in an electric field. Such polarizability can promote the adsorption of cysteine molecules on a metal interface [41,42,43].

### 3.4. Corrosion Inhibition Mechanisms

**Figure 16:**
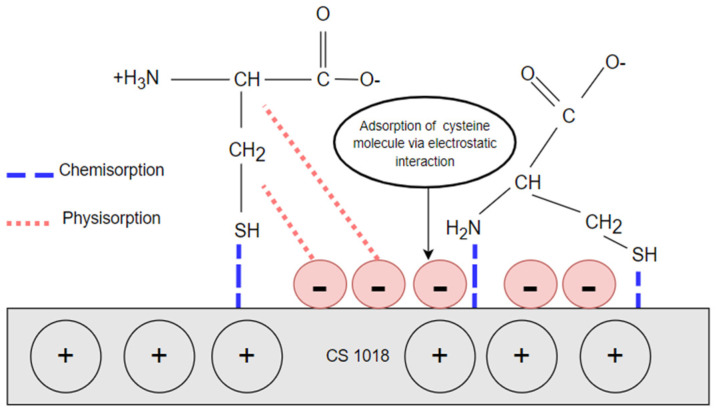
Proposed inhibition mechanism of cysteine.

In the aqueous amine-CO_2_ system, the corrosion process involves the oxidation of iron (Fe) (reaction 12) and reductions in oxidizing species (reactions 13–14). Bicarbonate ion (HCO_3_^−^) and water (H_2_O) are the primary oxidizing species which contribute to the formation of corrosion products, including iron carbonate (FeCO_3_) and iron hydroxide carbonate (Fe_2_(OH)_2_CO_3_) (reactions 15–16) [44]. From all of the obtained results and post-analysis, it can be seen that cysteine functioned by primarily reducing the iron oxidation rate. Its inhibition effectiveness is speculated to be attributed to the presence of mercapto (-SH) and amino (-NH_3_) groups, having a high affinity towards adsorption on metal surfaces [45]. As illustrated in Figure 16, cysteine interacted with the metal surface through a combination of both physical and chemical adsorption. The physisorption may occur by a electrostatic interaction of cysteine zwitterion molecules and carbon steel [46]. The chemisorption may be developed by forming a chemical bond between the lone pair of S and N atoms and the unoccupied d-orbital orbitals of carbon steel [47,48,49], thus forming a cysteine complex on the metal interface (Reaction 17).

Fe ↔ Fe^2+^ + 2e^−^ (12)

2HCO_3_^−^ + 2e^−^ ↔ H_2_ + 2CO_3_^2−^ (13)

2H_2_O + 2e^−^ ↔ H_2_ + 2OH^−^ (14)

Fe^2+^ + CO_3_^2−^ ↔ FeCO_3_ (15)

2Fe^2+^ + CO_3_^2−^ + 2H_2_O ↔ Fe_2_(OH)_2_CO_3_ (16)

Fe^2+^ + Cysteine ↔ Fe-Cysteine complex (17)

## 4. Conclusions

Cysteine showed great promise for becoming an alternative eco-friendly corrosion inhibitor to the commercial inorganic inhibitors that are typically used for absorption-based carbon capture plants. Its inhibition performance varied with the cysteine concentration, solution temperature, and flow condition, and could remain effective for the entire range of a plant’s operating conditions. Further, 500 ppm of cysteine was the optimum concentration, yielding inhibition efficiencies of up to 99% at 80 °C and 120 °C under dynamic flow conditions for when carbon steel was fully exposed to the solution. Its efficiency was reduced to the range of 80% when the carbon steel was exposed to a vapor phase. Cysteine functioned as an anodic-type corrosion inhibitor. It underwent spontaneous, endothermic adsorption which developed a sulphur-containing layer adjacent to the electrical double layer, improved the double layer resistance and strengthened the capacitor property of the carbon steel. The cysteine adsorption took place with both physisorption and chemisorption mechanisms, following the Langmuir isotherm model. 

## Figures and Tables

**Table 1:** Environmentally friendly properties [9,10,11].

	Chemical	LD_50_ (mg/kg)—Oral—Rat	Biodegradability	Bioaccumulation (as log *pow*)
Tested corrosion inhibitors	Cysteine (Cys)	5850	98% Readily biodegradable—After 28 days in aerobic environment.	−3.05
CO_2_ absorption solvents	Monoethanolamine (MEA)	1720	Readily biodegradable. In the strum test after 28 days, 91% of the MEA degrades.	−1.31
	Diethanolamine (DEA)	780	Depending on the soil conditions, biodegradable in soil.	−1.43
	Methyl diethanolamine (MDEA)	1945	After 28 days in activated sludge, it does not biodegrade, but it degraded more than 96% after 40 days in a continuous-flow study where the inoculum had time to acclimate.	−1.50
Inorganic corrosion inhibitors	Sodium metavanadate	183	(For Inorganic substance-Methods used for determining biodegradability are not applicable.)	Toxic to aquatic life with long lasting effects
	Copper carbonate	159		Very toxic to aquatic life
	Vanadium pentoxide	10		Toxic to aquatic life with long lasting effects
	Nickel (II) sulfate hexahydrate	362		May cause long term adverse effect in the aquatic environment.

**Table 2:** Calculated activation parameters.

System	*E_a_* (kJ mol^−1^)	Δ*H_a_* (kJ mol^−1^)	Δ*S_a_* (J mol^−1^ K^−1^)
Blank	30.28	27.44	−174.05
500 ppm Cysteine	9.86	7.10	−268.51

**Table 3:** Weight loss results.

Specimen	Corrosion Rate (mmpy)		Inhibition Efficiency (%)
	Blank	500 ppm Cysteine	
M1-Full immersion (dynamic-liquid phase)	3.47	0.30	91.37
M2-Full immersion (static-liquid phase)	1.74	0.18	89.63
M3-Partial Immersion (static -liquid & vapor phase)	1.12	0.02	98.60
M4-No immersion (static-vapor phase)	0.08	0.02	81.12

## Data Availability

No additional data were created.
